# EphA2 sustains the adaptive response of colorectal organoids to chemotherapy

**DOI:** 10.3389/fcell.2026.1833389

**Published:** 2026-06-12

**Authors:** Veronica Gatti, Gabriella Teresa Capolupo, Chiara Taffon, Andrea Marra, Giuseppe Perrone, Gianluca Masciana’, Marco Caricato, Mario Cioce, Vito Michele Fazio

**Affiliations:** 1 Laboratory of Molecular Medicine and Biotechnology, Department of Medicine, University of Campus- Biomedico of Rome, Rome, Italy; 2 Institute of Translational Pharmacology, National Research Council of Italy (CNR), Rome, Italy; 3 UOC Chirurgia Colorettale, Fondazione Policlinico Universitario Campus Bio-Medico, Rome, Italy; 4 Research Unit of Surgery, Department of Medicine and Surgery, Università Campus Bio-Medico, Rome, Italy; 5 Research Unit of Anatomical Pathology, Department of Medicine and Surgery, Università Campus Bio-Medico di Roma, Rome, Italy; 6 Anatomical Pathology Operative Research Unit, Fondazione Policlinico Universitario Campus Bio-Medico, Rome, Italy

**Keywords:** ALDH, chemoresistance, CRC, DTP, EphA2, patient-derived-organoids

## Abstract

**Introduction:**

EphA2 is highly expressed in colorectal cancer (CRC), and high EphA2 expression indicates a worse prognosis. We investigated EphA2 dynamics in a clinically relevant model: CRC patient-derived organoids (PDOs) treated with chemotherapy.

**Methods:**

We evaluated the number of EphA2-expressing cells and the Aldehyde dehydrogenase activity by flow cytometry, and analyzed EphA2 protein levels and phosphorylation status using Zn-Phos-tag gels and indirect ELISA. We employed siRNA to deplete the PDO cells of EphA2.

**Results:**

EphA2-positive cells form a stable subpopulation in organoid cultures that persists after oxaliplatin treatment. Phosphorylation of EphA2 at Ser897 increases with treatment and correlates with higher EphA2 levels. Silencing EphA2 or reducing Ser897 phosphorylation decreases organoid formation, suggesting chemosensitization. Some EphA2-positive cells show increased ALDH activity after chemotherapy, and EphA2-ALDH1A3 interaction has prognostic value in CRC.

**Discussion:**

Here, we discovered that EphA2-positive cells constitute a persistent, ALDH-positive cell subpopulation in CRC-PDOs that withstood exposure to oxaliplatin (OXA) and 5-fluorouracil (5-FU). The enforced suppression of EphA2 or diminished Ser897 stress results in a chemosensitization effect. This sheds further light on the role of EphA2 in the adaptive stress response of CRC.

## Background

Colorectal cancer (CRC) is a leading cause of cancer-related death worldwide (Roshandel et al., 2024). Oxaliplatin- and 5-fluorouracil (5-FU)-based chemotherapy is a standard component of treatment for patients with advanced CRC (Fadlallah et al., 2024). As with many solid cancers, the emergence of resistance is a major therapeutic challenge and may involve cell-autonomous and non-cell-autonomous mechanisms (Kallberg et al., 2023; Oh et al., 2025). Thus, adaptive stress-induced resistance is a key driver of CRC progression and an important area of investigation. EphA2 (Ephrin type-A receptor 2) belongs to the Eph kinase family, which is the largest subfamily of the receptor tyrosine kinase superfamily. The Ephrin receptor-ligand system enables bidirectional signaling, a pivotal mechanism of tissue patterning under physiological conditions (Pasquale, 2008). EphA2 has long been associated with cancer initiation and progression (Pasquale, 2024). High levels of EphA2 predict tumor progression and poor prognosis in many solid tumor settings, including CRC (Herath and Boyd, 2010; Pasquale, 2024). Specifically, EphA2 expression has been associated with risk factors and poor overall survival (OS) in 10 out of 33 neoplasms (Li et al., 2024). This encompasses the acquisition of invasive and metastatic abilities (Wilson et al., 2021). EphA2 promotes self-renewal, proliferation, and invasion, and acts as a driver of stemness in glioblastoma and lung cancer stem-like cells (Binda et al., 2012; Hamaoka et al., 2016; Song et al., 2014). This protein is also highly expressed in OCT4-positive, undifferentiated, pluripotent stem cells (Intoh et al., 2024).

Recent and past studies have supported a dichotomous view of EphA2 signaling: a ligand-stimulated, tyrosine phosphorylation-mediated vs. a ligand-independent serine transphosphorylation model, with the former supporting a pro-tumorigenic effect (Kaibori et al., 2020; Singh et al., 2015; Zhou and Sakurai, 2017). The interplay between tyrosine phosphorylation and the protumorigenic activities of EphA2 is complex and may be mediated by the phosphorylation of EphA2’s serine residues, increased EphA2 levels, and downstream ERK signaling, as demonstrated in glioblastoma (Hamaoka et al., 2016; Hamaoka et al., 2018).

However, tumor-specific mechanisms likely exist, and ligand-mediated and independent EphA2 phosphorylation may be context-specific. More recently, changes in the EphA2 oligomerization state have been identified as crucial for the receptor’s oncogenic function. More specifically, the oligomerization state of the receptor depends on its expression levels *in vitro* and *in vivo* and is influenced by local cues, such as membrane cholesterol content (Sahoo et al., 2025; Wirth et al., 2024). Quantitative proteomic analyses have shown that EphA2 is enriched in exosomes from drug-resistant cells (Fan et al., 2018; Gao et al., 2021). This suggests that increased EphA2 content may be related to and confer drug resistance. The way EphA2 contributes to drug resistance, the ultimate driver of cancer progression, remains unexplored. A related question concerns the modulation of EphA2 function under stressful conditions, such as during chemotherapy treatment. The functioning of EphA2 can be modulated by kinase phosphorylation in the absence of a ligand. For example, stress- and MAPK-mediated phosphorylation of EphA2 has been described in breast, lung, and ovarian cancer cells treated with platinum-containing compounds and ionizing radiations (Kaminskyy et al., 2021; Moyano-Galceran et al., 2020; Zhou et al., 2023).

In CRC, Mitra et al. found that the hyperactivation (phosphorylation) of EphA2 (and c-Met) in patients treated with 5-fluorouracil (5-FU)-containing chemotherapy correlated with lower disease-free survival (Mitra et al., 2020). Additionally, glioblastoma cell proliferation is promoted by overexpression of wild-type EphA2 (Hamaoka et al., 2016). EphA2 membrane expression appears to mark cell subpopulations within mouse CRC tumors that have a distinct microRNA repertoire and possibly distinct biological and therapeutic characteristics (De Robertis et al., 2018). Altogether, this raises the possibility that EphA2 increased activation or expression may influence specific protumorigenic properties, including drug resistance. Patient-derived organoids (PDOs) are self-organizing 3D structures that mimic the cellular heterogeneity of the source tissue. Therefore representing clinically relevant *in vitro* models (Qu et al., 2021; Thorel et al., 2024). The ability to form organoids is regarded as a functional feature of healthy tissue stem cells, as it demonstrates fundamental properties such as self-renewal and differentiation (Andersson-Rolf and Clevers, 2026). In cancer settings, residual organoid-forming ability after a treatment may indicate persistence of drug-tolerant cells with stem-like features (He et al., 2024). We have demonstrated that drug response and resistance to therapy correlate with organoid-forming ability in CRC, metastatic breast cancer, and mesothelioma (Cioce et al., 2023; Cioce et al., 2024; Donzelli et al., 2022). Here, by coupling EphA2 expression detection in oxaliplatin- and 5-fluorouracil-treated PDOs, we set out to investigate EphA2’s role in mediating an adaptive response to oxaliplatin-induced stress in clinically relevant CRC models.

## Results

### EphA2-positive (EphA2^pos^) cells are present in CRC patient-derived organoids (PDOs) and persist over passaging

**FIGURE 1:**
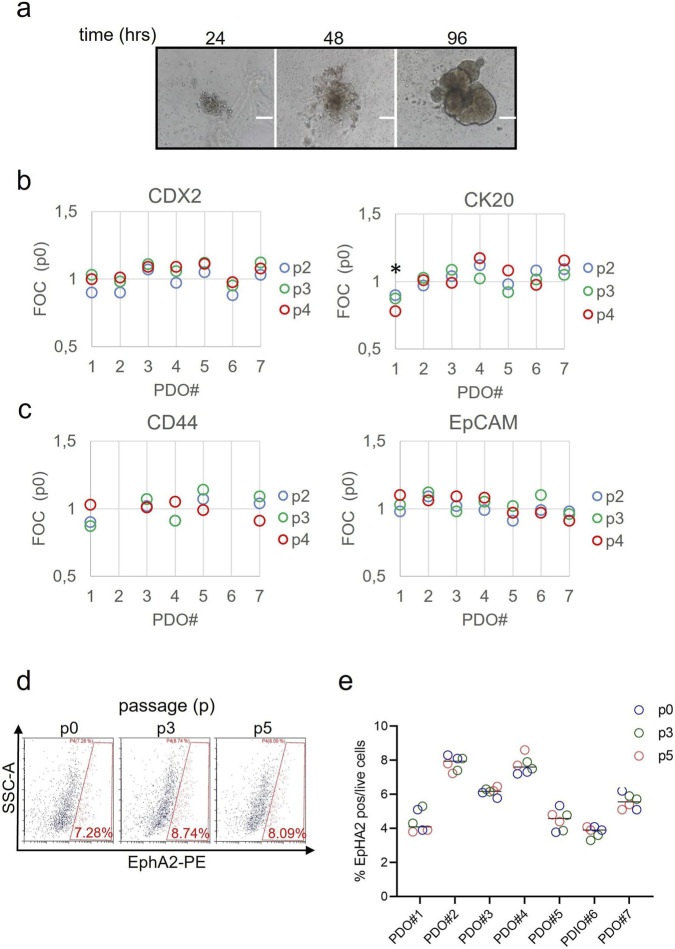
EphA2^pos^ cells are similarly represented in CRC specimens and matched PDOs, and they persist over PDO passaging. Patient-derived organoids (PDOs) were obtained from seven CRC specimens and characterized for their expression of mRNA markers immediately after disaggregation (p0) and after two to four passages (p2, p3, p4). **(a)** Representative micrographs show PDOs growing over time after disaggregation, forming convoluted structures. Scale bar: 200 μm **(b,c)**. Graphs showing the relative expression levels of CDX2, CK20, CD44, and EpCAM mRNA, as detected by QRT-PCR, in PDO cultures at passages zero (reference: p0), two (p2), three (p3), and four (p4). The average of triplicate experiments for each passage is reported. Values are expressed as fold change over control (p0, set at 1). No significant changes in the expression of the markers were recorded, except where indicated by the asterisk (*). *p < 0.05 vs. passage 0. **(d)** Representative dot plots showing the percentage of EphA2-positive cells/live cells evaluated by flow cytometry in PDO#4 at p0 and after serial passaging (p3 and p5). Gating was determined based on staining with an irrelevant isotype antibody. **(e)** The histogram shows the percentage of EphA2-positive cells at p0, p3, and p5 in all seven PDO cultures. No statistically significant differences were observed at p0, p3, or p5 (p > 0.05). Statistics: *p < 0.05; **p < 0.01. Absence of asterisk denotes no statistically significant difference.

We set up patient-derived organoid (PDO) cultures (n = 7) from CRC patient specimens (right side, n = 4; left side, n = 3; see Supplementary Table S1). The organoids grew into 3D aggregates that became increasingly complex over time, forming convoluted structures (Figure 1a). To validate the PDOs’ ability to represent the originating specimens, we evaluated the expression levels of CDX2, CK20, CD44, and EpCAM CRC markers (Bayrak et al., 2012; Liu et al., 2018; Werling et al., 2003), at passage 0 (p0, immediately after mechanical and enzymatic disaggregation) and passage 2,3,4 (p2,p3,p4) after an average of 35 days of culturing (28–42 days). This revealed that the expression of all four markers was maintained across passaging, except for modestly decreased CK20 expression in PDO#1 at passages 2–4, which reached statistical significance (p < 0.05) (Figures 1b,c). Thus, the organoid cultures exhibited an acceptable degree of similarity to their originating specimens. We evaluated the number of EphA2^pos^ cells in CRC PDOs and found that they were detectable in all organoids and persisted through sequential passaging (Figure 1d). No statistically significant difference was observed in the PDO cultures at p0, p3, or p5 (p > 0.05) (Figure 1e).

### EphA2^pos^ cells persist in oxaliplatin-resistant patient-derived organoid (PDO) cultures

**FIGURE 2:**
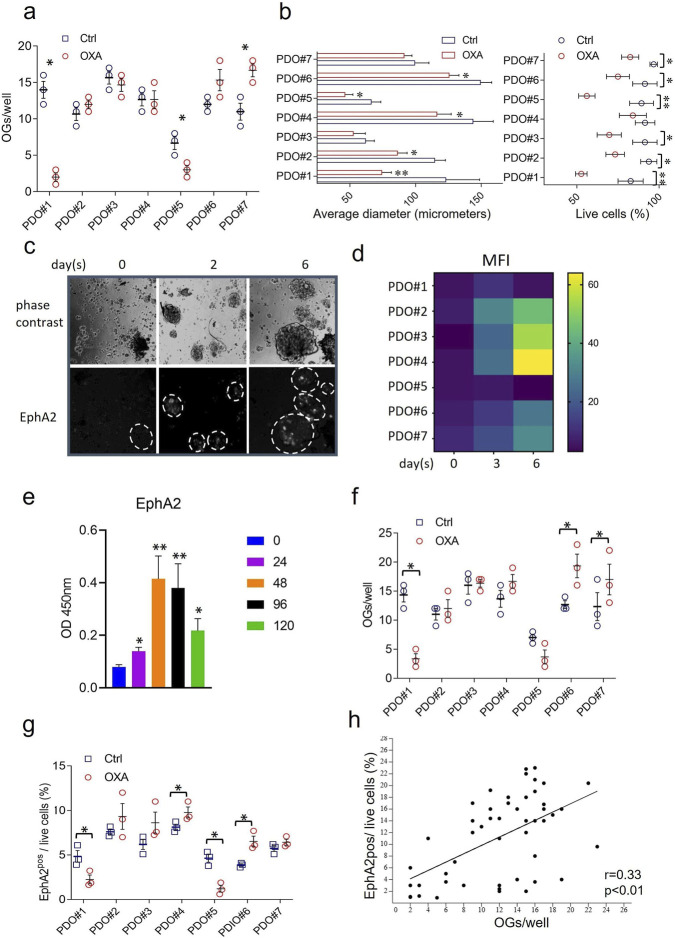
EpHA2^pos^ cells persist in oxaliplatin-treated CRC PDOs. **(a)** PDOs one to seven were treated with Ctrl (saline) or 5 μmol/L OXA for 96 h (with drug washout at 24 h). **(a)** The number of newly formed organoids after disaggregation and at the end of the treatment was recorded (and defined as OFA; see text). **(b)** Left panel. The average diameter of the formed organoids at the end of the treatment was assessed with ImageJ on phase contrast images. Right panel. Percentage of live cells evaluated by flow cytometry in disaggregated PDO treated with Ctrl or OXA. **(c)** Representative phase-contrast images of PDO #4 treated with 5 μM OXA for the indicated days, then fixed with PFA and stained with an anti-EpHA2 antibody at each indicated time point. The relative position of the PDOs is indicated by dashed circles. **(d)** Median fluorescence intensity (MFI) for EphA2-positive cells in PDOs #one to seven treated as in **(c)**, calculated after selecting positive cells as ROI. **(e)** Histogram reporting the levels of EphA2 in the whole cell lysate (WCL) of PDOs harvested at the indicated times after OXA treatment, detected by ELISA. **(f)** Graph reporting the organoid-forming ability (reported as OG/well) of PDOs #one to seven treated with OXA for 21 days **(g)**. Percentage of EphA2-positive cells/live cells after 21 days of oxaliplatin treatment, assessed by flow cytometry **(a–g)** Mean +SEM of triplicate measurements. **(h)** Linear correlation analysis between the number of EphA2-positive cells and the formed organoids per well, after chronic Ctrl and OXA treatment. Data from quadruplicate measurements (r = 0.3345 and p = 0.0073). Statistics: *p < 0.05; **p < 0.01. Absence of asterisk denotes no statistically significant difference.

Given EphA2’s possible role in mediating the response to chemotherapy, we evaluated changes in the EphA2^pos^ subpopulation within the PDOs challenged with oxaliplatin (OXA) or saline (Ctrl) for 96 h at a pharmacologically relevant concentration (0–10 μmol/L) with a drug washout after 24 h. We evaluated the response of the organoid cultures by recording the number of newly formed organoids, the average diameter, and the percentage of live cells after disaggregation and at the end of the treatment (Figures 2a,b). Regarding the former parameter, five PDO cultures showed resistance to OXA, whereas PDO#1 and PDO#5 were sensitive (Figure 2a). The average maximum diameter and the percentage of live cells decreased concomitantly in the treated samples (Figure 2b), suggesting that different cell subpopulations with varying sensitivities to the drug are present in the OGs and that the main difference between the OGs lies in their ability to reform organoids after treatment.

We assessed the number of EphA2^pos^ cells in fixed PDOs treated with 5 μM OXA for 0–5 days. This revealed an increase in positive cells over time, persisting up to 6 days after drug washout (Figures 2c,d). Concomitantly, the median fluorescence intensity (MFI)/cell steadily increased over time upon OXA challenge (Figure 2d). This was common to all PDO cultures except PDO#1 and PDO#5 (Figure 2d). An ELISA assay performed on whole-cell lysates (WCL) of the treated PDO cultures for 0–120 h confirmed increased EphA2 protein levels, supporting the observed increase in MFI (Figure 2e).

### EphA2^pos^ cells persist after chronic oxaliplatin (OXA) treatment

Since the persistence of EphA2^pos^ cells at steady state (Figure 1e) and after acute OXA treatment (Figures 2c–e) indicates a stable population, we evaluated the number of EphA2^pos^ cells after OXA treatment (5 µM) for 21 days, with drug renewal every 96 h. First, we calculated the number of organoids per well after 21 days of treatment. We found that after chronic OXA treatment, the number of newly formed organoids was unchanged in most PDO cultures, except for PDO#1 and PDO#5 (Figure 2f), consistent with the observations in Figure 2a. Concomitantly, we found that the number of EphA2^pos^ cells within chronically treated PDOs closely mirrored the changes observed in Figure 2f (Figure 2g), suggesting a relationship between the number of EphA2^pos^ cells and the residual organoid-forming capacity. Linear correlation analysis confirmed such a relationship (Figure 2h, r = 0.334, p < 0.01). Similar results were obtained with 5-FU treatment (Supplementary Figures S1a–c), suggesting that what was observed may be part of a broader adaptive response to treatment. This prompted us to analyze EphA2 total protein levels in PDO-derived lysates treated with OXA.

### The organoid forming ability upon oxaliplatin treatment is affected by total EphA2 levels

**FIGURE 3:**
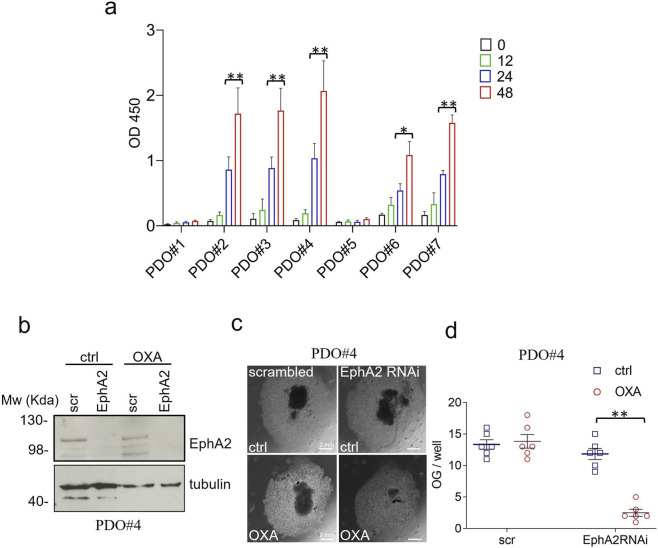
OXA treatment increases the levels of EphA2 in CRC PDOs. **(a)** The levels of EphA2 total protein after OXA treatment were detected by ELISA at the indicated times. Please note that differences were statistically significant at 24 and 48 h After treatment. **(b)** Western blotting of whole-cell lysates from PDO#4, which were transfected with scrambled or EphA2-directed siRNA, treated with vehicle or OXA, and stained with an anti-EphA2 antibody. Tubulin was used as a loading control. **(c)** Representative micrographs of PDO#4 treated with scrambled or EphA2 RNAi in the presence or absence of OXA. **(d)** Quantitative evaluation of formed organoids/well). Statistics: *p < 0.05; **p < 0.01. Absence of asterisk denotes no statistically significant difference.

We performed indirect ELISA assays using whole-cell lysates from all seven PDO cultures to evaluate total EphA2 protein levels after OXA treatment. This showed that a significant increase in EphA2 was observed across all PDOs previously classified as resistant to OXA. (Figure 3a). To evaluate the functional contribution of increased EphA2 levels to organoid-forming efficiency, we transfected the PDO#4 with either EphA2-or Ctrl-siRNAs 24 h before challenging with vehicle or OXA for an additional 72 h. We validated the RNAi-mediated EphA2 knockdown by Western blotting 48 h after transfection (Figure 3b). At steady state, the number of formed organoids was slightly attenuated upon EphA2 knockdown compared to scrambled-transfected cultures (Figures 3c,d). Conversely, it significantly decreased in EphA2 RNAi-treated PDO cells upon OXA treatment (Figures 3c,d), suggesting that EphA2 abundance may be relevant to the organoids’ adaptive response to OXA. Thus, altering EphA2 levels affects the residual organoid-forming ability of treated CRC PDOs, resulting in a chemosensitizing effect.

### EphA2 is Ser897-phosphorylated in oxaliplatin-treated CRC PDOs

**FIGURE 4:**
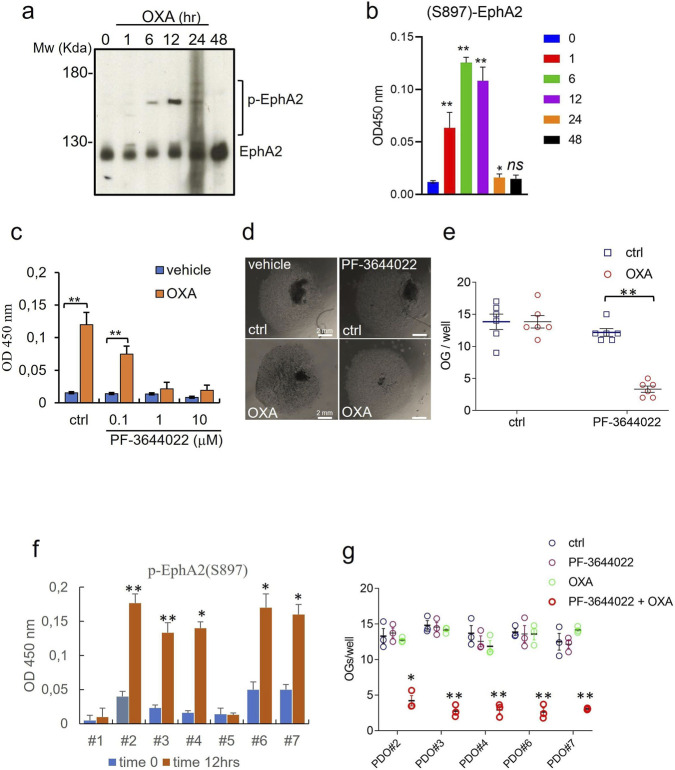
OXA treatment increases the Ser897 phosphorylation of EphA2 in an MK2-dependent way. **(a)** Western blotting with an EphA2 antibody of whole cell lysate (WCL) from PDO#4, treated as indicated with 5 μmol/L OXA, and subjected to Zn-Phos-Tag SDS-PAGE gel separation. **(b)** ELISA assay to detect the Ser897-EphA2 levels in time after OXA treatment. **(c)** Levels of Ser-897 EphA2 after treatment with the MK2 inhibitor PF-3644022, in the presence of vehicle or OXA, detected by ELISA **(b,c)** Mean ± SEM of quadruplicate detections. **(d)** Representative micrographs of PDO #4 treated with vehicle or PF-3644022 in the presence or absence of OXA. **(e)** Quantitative evaluation of organoid-forming ability (OG/well) for the PDO#4 treated as in **(d)**. Mean ± SEM of sextuplicate measurements. **(f)** The phosphorylation of Ser897-EphA2 is common to OXA-resistant PDOs. **(f)** ELISA assay to detect the levels of Ser897-EphA2 in the seven PDOs treated with OXA for 12 h. **(g)** Treatment with the MK2 inhibitor PF-3644022 blunts PDO resistance to OXA. The number of OG/well was evaluated after the indicated in the OXA-resistant PDOs **(f,g)** Mean ± SEM of triplicate measurements. Statistics: *p < 0.05; **p < 0.01. Absence of asterisk denotes no statistically significant difference. Please note that in **(g)** the statistics are reported for OXA vs. PF-364402 + OXA.

To investigate the EphA2 posttranslational status in OXA-treated cells, we ran whole cell lysates from PDO#4 (treated or untreated with 5 μM OXA for up to 48 h) through a Zn-Phostag gel. This revealed post-translationally modified, EphA2-positive forms when the filter was decorated with an anti-EphA2 antibody (Figure 4a). This occurred 6 h after OXA treatment, peaked between 12 and 24 h after treatment, and then decreased after 48 h (Figure 4a). Furthermore, since serine 897 is a primary site of EphA2 phosphorylation under stress; Moyano-Galceran et al., 2020), we used an indirect sandwich ELISA to determine whether EphA2 phosphorylation levels increased after OXA treatment in PDOs. The results showed that the Ser897 signal increased after treatment, with kinetics similar to those observed in Figure 4a (Figure 4b), suggesting that Ser897 of EphA2 is an oxaliplatin-sensitive phosphorylation modification in CRC PDOs. EphA2 phosphorylation in OXA-treated PDOs may be mediated by MAPK-activated protein kinase 2 (MK2). MK2 has been shown to mediate EphA2 phosphorylation at Ser897 in response to cisplatin or high osmotic stress (Zhang et al., 2025; Zhou et al., 2023). We evaluated the effect of pretreatment with an MK2 inhibitor on OXA-induced EphA2 Ser897 phosphorylation. We pretreated PDO#4 with PF-3644022 (a reversible, ATP-competitive MK2 inhibitor) at three different concentrations for 90 min. Then, we evaluated the Ser897 phosphorylation by ELISA 6 h after adding oxaliplatin. This revealed that the MK2 inhibitor had little effect on Ser897-EphA2 at steady state (p > 0.05), with a significant effect observed when the pretreatment was followed by OXA (Figure 4c). To explore the functional consequences of attenuated EphA2 Ser897 phosphorylation, we evaluated the residual organoid-forming ability of PDO#4 pretreated or not with PF-3644022 before the addition of the vehicle or OXA. In the absence of OXA, the MK2 inhibitor marginally affected the number of organoids formed by the treated cells. Conversely, a striking effect was observed when OXA was co-administered with PF-3644022, resulting in a reduction of more than 80% in the number of formed organoids (Figures 4d,e). These results suggest that MK2 mediates the increase in EphA2 Ser897 phosphorylation, thereby affecting the residual organoid-forming ability after treatment.

Additional validation in PDOs. ELISA assay revealed that Ser897 phosphorylation was detectable in vehicle-treated PDOs and increased upon OXA in all the resistant PDOs. On the other hand, in the OXA-sensitive PDO#1 and PDO#5, a very weak, if any, signal for Ser897 phosphorylation could be observed, and this latter was not increased by OXA treatment (Figure 4f). This was strictly paralleled by the increase in total EphA2 levels in all but PDO#1 and PDO#5 (Figure 3a). Next, we tested whether MK2 inhibition could affect the PDO cultures’ response to OXA. When assessing the number of reformed organoids per well, we found that this latter was unaffected in vehicle-treated cells (Figure 4g). On the other hand, the PF-3644022 increased the sensitivity of the resistant PDOs to OXA (p < 0.05) (Figure 4g). Since it has been shown that Ser897 can be phosphorylated by AKT (Miao et al., 2009), we evaluated whether AKT could also mediate phosphorylation of Ser897 in OXA-treated PDOs. ELISA assays revealed that the Ser897 phosphorylation was only slightly affected by the AKT inhibitor MK-2206 (5microMol/L) in vehicle-treated cells (p < 0.05), while the phosphorylation of the same residues induced by OXA treatment was unaffected (Supplementary Figure S2), supporting a specific role for MK2 in mediating EphA2 Ser897 phosphorylation in OXA-treated cells.

### The ALDH^high^ and EphA2^pos^ cell subpopulations partially overlap in PDOs treated with chemotherapy

**FIGURE 5:**
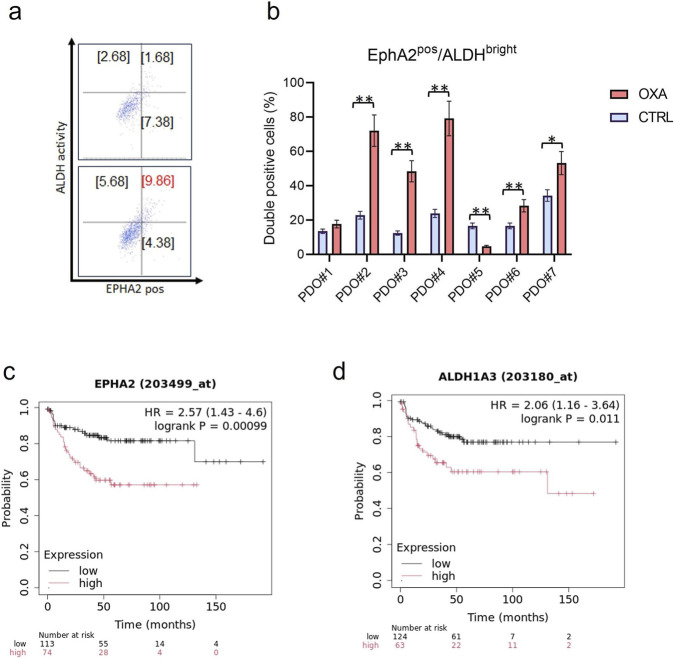
EphA2^pos^/ALDH^high^ double-positive cell subpopulations increase after OXA treatment in CRC PDOs **(a)**. CRC PDOs were treated with OXA for 24 h. 48 h later, the cells were disaggregated and analyzed by flow cytometry for EphA2 expression and aldehyde dehydrogenase (ALDH) activity. The percentage of ALDH^high^ cells was calculated as background staining after treatment with the ALDH inhibitor DEAB. **(b)** Graph reporting the average of two independent experiments performed as indicated in **(a) (c,d)** EphA2 and ALDH1A3 expression are correlated in advanced (stage 3–4) CRC patients in the MSI CMS. Kaplan-Meier curves were generated for CRC TGCA patients, and Pearson correlations were calculated using KM-plotter.com. Statistics: *p < 0.05; **p < 0.01. Absence of asterisk denotes no statistically significant difference.

In several cancer settings, including colorectal cancer (CRC), resistance to chemotherapy has been associated with the emergence of cells with high aldehyde dehydrogenase (ALDH) activity, known as ALDH^high^ cells and endowed with stem-like features (Lavudi et al., 2024; Xia et al., 2023; Kozovska et al., 2018; Chen et al., 2015). Additionally, inhibition of ALDH activity has been shown to sensitize CRC cells to chemotherapy (Durinikova et al., 2018; Kozovska et al., 2018). Given the ability of EphA2^pos^ cells to survive oxaliplatin (OXA) treatment, we explored whether these cells would exhibit high ALDH activity. Flow cytometry-based detection of ALDH activity and EphA2 expression revealed that a small percentage of double-positive cells existed in the PDOs at steady state. Interestingly, the percentage of EphA2^pos^ cells with ALDH activity remained stable or increased after OXA treatment, ranging from no change to a several-fold increase compared to vehicle-treated samples (Figures 5a,b). Similar results were obtained after 5-FU treatment (Supplementary Figure S3). Further supporting this finding, analysis of patients with advanced CRC from TGCA revealed significant associations between ALDH1A3 and EphA2 mRNA expression and relapse-free-survival (RFS), as shown by Kaplan–Meier plots (Figures 5c,d). Additionally, the two variables were significantly correlated (Pearson correlation coefficient: 0.295, p = 0.005, n = 184) in the Consensus Molecular subtype 1 (CMS1), characterized by microsatellite instability, inflammatory signatures, and shown to derive limited benefit from chemotherapy-based regimens (like FOLFIRI) (de Back et al., 2024; Dienstmann et al., 2017). No significant correlation was observed in the remaining CRC consensus subtypes (data not shown). Thus, a fraction of ALDH^high/^EpHA2^pos^ cells persists in OXA-treated specimens, and this may correlate with increased PDO resistance to OXA and 5-FU.

## Discussion

Intra-tumor heterogeneity (ITH) is a feature of solid tumors and CRCs that impairs cancer progression (Bowes et al., 2022; Li et al., 2022). It is increasingly clear that distinct cell subpopulations within tumors have specific functions ranging from tumor initiation to adaptive resistance to stress, all of which contribute to tumor recurrence (Cioce et al., 2021b; Kallberg et al., 2023; Kumar et al., 2026; Sinai Borker et al., 2025). Here, we found that EphA2^pos^ cells represent a stable population in CRC PDOs that survived treatment with pharmacologically relevant doses of OXA and 5-FU. Increased levels of EphA2 are essential for the adaptive response to stress in these structures, and forced downregulation of EphA2 or reduced Ser897 phosphorylation decreases the organoid-forming ability (OFA)following oxaliplatin or 5-fluorouracil treatment. The residual organoid-forming ability is a predictive surrogate for cancer progression (Ishiguro et al., 2017; Thorel et al., 2024). We found that interfering with the increase in EphA2 levels after OXA treatment reduced the OFA of the treated specimens while leaving vehicle-treated CRC PDOs unaffected. Thus, EphA2 is more relevant under adaptive stress than under steady-state conditions for organoid formation. This is consistent with the prognostic value of EphA2 levels on RFS in CRC patients, which is more pronounced in patients who have undergone neoadjuvant treatment. Consistent with this, we found that EphA2^pos^ cells exhibited Aldehyde Dehydrogenase activity, a characteristic of stress-resistant cell, stem-like cell subpopulations. Consistent with this finding, the number of EphA2^pos^/ALDH^high^ cells increased in most treated CRC PDOs after a chemotherapy challenge (OXA or 5-FU). We observed a significant correlation between oxaliplatin-mediated Ser897 phosphorylation and PDO cultures’ sensitivity to oxaliplatin. Altogether, this raises the possibility that EphA2 may mark the so-defined Drug-Tolerant-Persister -cells (DTP) (Wang et al., 2025; Pu et al., 2023), demonstrated in CRC specimens (Nojima et al., 2025; Tape, 2024) and shown to be endowed with ALDH1A3 expression (Chen et al., 2024; Dhanyamraju et al., 2022).

The two organoid cultures with the lowest Ser897 phosphorylation exhibited the highest sensitivity to oxaliplatin (Figure 3). One limitation of this study is that we can only provide correlative evidence of an increase in phosphorylated Ser897 in EphA2 following chemotherapy treatment (Figure 4) and of a rise in EphA2 levels (Figure 3a). Further research is needed to investigate the mechanistic details of the relationship between Ser897 phosphorylation and increased EphA2 levels. One possible hypothesis is that, following Ser897 phosphorylation, the increase in EphA2 may alter the oligomeric composition of EphA2 complexes, known to be influenced by protein levels (Wirth et al., 2024). This would affect ALDH activity, leading to increased detoxification capacity and increased survival of cancer cells. In mesothelioma, we and others have demonstrated that this occurs transcriptionally via STAT3 and NFkB (Canino, Luo et al., 2015). The existence of a chemotherapy-related double-positive population of ALDH^high^ and EphA2^pos^ cells, and the correlation between EphA2 and ALDH1A3 levels in the MSI-H, CMS1 CRC subgroup, would be consistent with the above considerations. Our data are also reminiscent of recent studies in different tumor settings, where RNAi-mediated depletion of EphA2 in non-small cell lung cancer (NSCLC) lines decreased the ALDH-positive cell population and multicellular tumor spheroid formation. This was supported by a positive association between EphA2 and ALDH expression in lung cancer tissue microarrays (Song et al., 2014).

Another limitation of this study is that PDO cultures retain tumor microenvironment (TME) components to a limited extent (Yao et al., 2025). This experimental system may not allow assessment of whether TME-originated signaling mediates EphA2 modulation in treated samples. In support of this, exosome-mediated delivery of EphA2 signaling has recently been shown to confer protumorigenic properties to neighboring cells (Fan et al., 2018; Gao et al., 2021), providing provocative evidence in this regard. Last but not least, we acknowledge that in this experimental setting, we did not assess the presence of untransformed cells in the PDO cultures. In detail, although it is unlikely that untransformed cells would survive 21 days of treatment with Oxaliplatin and 5FU, we cannot rule out the possibility that some untransformed colorectal cells are mixed with the transformed ones. In this instance, whether the mixed nature of the PDO cultures and the drug-induced senescence of untransformed cells (Liang et al., 2025) may contribute to the observed resistant phenotype (Liu et al., 2024) remains an unexplored possibility and a limitation of this study.

Our findings suggest that reducing EphA2 Ser897 phosphorylation in chemotherapy-treated PDOs could counteract their resistance to oxaliplatin and 5-fluorouracil (5-FU). Since PDOs are clinically relevant models, this may have translational potential. As of this writing, PF-3644022 has not yet been used in phase 3 clinical trials. However, several FDA-approved MEK inhibitors may be suitable for clinical testing in oncology and non-oncology settings (https://clinicaltrials.gov/study/NCT06648434).

Furthermore, high EphA2 levels could help identify potential responders/non-responders to first-line chemotherapy, which is still used in the MSI-H setting despite the approval of ICIs for first-line treatment. Thus, our findings elucidate EphA2-driven adaptive stress response mechanisms, with potential relevance for patient stratification, particularly in the MSI-H subgroup.

## Methods

### Patient-derived organoid cultures (PDOs)

CRC specimens were obtained from the Fondazione Policlinico Universitario Campus Biomedico according to the Ethical Committee of the Università Campus Biomedico, Rome (code CR-CAF-MIC). All patients provided written informed consent, and samples were anonymized before they were released. This data does not evaluate associations with sex or gender.

Patient-derived organoids (PDOs) were obtained according to published protocols with no modifications (Cioce et al., 2023; Sato et al., 2011). Briefly, right and left colon adenocarcinoma biopsies were minced into<1 mm pieces, and enzymatically and mechanically digested with hyaluronidase/collagenase (Stem Cell technologies, Vancouver, CA) for 1 h at 37 °C with occasional swirling. Cells freed from tissue were filtered through a 100 mM mesh (Starlab, Milan, Italy) and suspended in extracellular matrix drops (Geltrex LDEV-Free reduced growth factor-basement membrane extract, Thermofisher, Waltham, MA, United States). The number of cells included in Geltrex was kept constant across all PDOs (200 live cells/drop). The IntestiCult™ Plus Balance Organoid Growth Medium (Stem Cell Technologies, Vancouver, CA) was added to the jellified drops according to the manufacturer’s instructions. The PDO cultures were passaged every 5–7days by mechanical-enzymatic disaggregation as mentioned.

### Validation of PDO cultures

The expression of CK20, CDX2, CD44, and EpCAM was assessed by QRT-PCR in PDO cultures at passage 0 (immediately after mechanical and enzymatic disaggregation) and at passages two to four, after an average culture persistence time of 35 days (28–42).

### RNA extraction, cDNA synthesis, and qRT-PCR

Total RNA was extracted using the RNAeasy minikit (QIAGEN). The first-strand cDNA was synthesized with the High-Capacity RNA-to cDNA kit (ThermoFisher, Waltham, MA, United States). Gene expression was measured by real-time PCR using SYBR Green dye on a StepOne instrument (Thermo Fisher Scientific, Waltham, MA, United States). Human qPCR Primer Pairs were from Origene (Origene, Rockville, MD, United States).

### Reagents

Oxaliplatin, 5-FU, and the MK2 inhibitor PF-3644022 were from Cayman (Cayman Chemicals, Ann Arbor, Michigan, United States) and dissolved in DMSO. The AKT inhibitor MK2206 was from Selleckchem (Selleckchem, Houston, TX 77014 United States). In all experiments, the maximum DMSO concentration used as a control was ≤0.05%. A table with the reagents employed and their working dilutions is available as Supplementary Table S2.

### PDO Treatment

PDO were mechanically and enzymatically disaggregated to single cells and 500–1,000 live cells were plated into 24-well plates 24 h before starting treatments.

### Organoid forming ability (OFA) assay

The OFA was developed according to Bergin and colleagues (Bergin and Benoit, 2022) with some slight modifications. Briefly, mechanically and enzymatically disaggregated PDOs were filtered through a 30-μm mesh and plated into 30-μL extracellular matrix drops in sextuplicate wells at 100 live cells/drop. The number of newly formed organoids with a minimum average diameter of 50 μm was recorded after 7–10 days. The average diameter of the formed organoids after treatment was measured by collecting brightfield microscopy images (×4 objective) and employing ImageJ (https://imagej.net/ij/index.html) to measure the major diameter of the approximately ellipsoidal shape for each CRC organoid. Cell viability was assessed by flow cytometry by employing a Helix-NP Blue viability dye (Biolegend, San Diego, CA, United States).

### Immunostaining protocol

Briefly, the intact organoids were devoid of extracellular matrix using Organoid Recovery solution (The Well Bioscience Inc., NJ 08852, United States) according to the manufacturer’s instructions, and the mixture was allowed to pellet by low-speed centrifugation (300 rpm × 10 min). After that, the structures were fixed in 4% PFA, washed twice with PBS1X and gently seeded in PBS-1% BSA for the staining in 96 flat bottom dishes (Corning, NY, United States).

### Indirect immunofluorescence

PDO cultures were stained, unpermeabilized, with an Alexa Fluor 488-conjugated Monoclonal Mouse IgG_2A_ Clone # 371805 (R&D Systems, Minneapolis, MN, United States). A phase-contrast micrograph was recorded simultaneously to localize the stained organoids. Images were recorded with a Nikon Eclipse Ti microscope -based system (Nikon, Tokyo, Japan).

### Flow cytometry

Briefly, PDO-derived cells (after mechanical and hyaluronidase-collagenase digestion) were filtered through a 70 μm mesh (Starlab, Milan, Italy) and incubated on ice for 30 min with PE-conjugated mouse IgG2b anti-human EphA2 antibody (clone SHM16). A PE-IgG2b isotype control was used to gate out background staining (BioLegend, San Diego, CA, United States). Both antibodies were co-incubated with Human TruStain FcX™ (Fc Receptor Blocking Solution, Biolegend, San Diego, CA, United States) to minimize non-specific binding. Five minutes before analysis, Helix-NP Blue viability dye (BioLegend, San Diego, CA, United States) was added to aid in gating live cells. Cell populations were identified using a Cytoflex flow cytometer (Beckman Coulter Life Sciences, IN, United States).

### ELISA-based detection of total and Ser897 (EphA2)

Human EphA2 was detected by a solid-phase sandwich Enzyme-Linked Immunosorbent Assay (ELISA) (Thermo Fisher Scientific, Waltham, MA, United States, cat. #EH173RB), according to the manufacturer’s instructions. Ser897-phosphorylated EphA2 was detected by substituting the anti-EphA2 capture antibody of the former kit with a Phospho-EphA2 (Ser897) Polyclonal Antibody (Thermofisher, Waltham, MA, United States, cat# PA5-117243). Biotinylated detection antibody and Streptavidin-HRP reagent were used according to the manufacturer’s instructions. OD450 nm was recorded within 30 min from the reaction.

### Western blotting

Briefly, whole cell lysates from Matrigel-free PDO cultures (after enzymatic removal of Matrigel) were obtained in NP-40 buffer (150 mM sodium chloride, 1.25% IGEPAL CA-630, 50 mM Tris pH 8.0, 5% Glycerol, Protease and Phosphatase Inhibitor Cocktail (Merck Life Science, Milan, Italy). Anti-EphA2 antibody (Santa Cruz, Dallas, Texas, United States) was used to decorate the PVDF filter, and, as a loading control, anti-tubulin antibody (EPR-7951, Abcam, Cambridge, United Kingdom) was used for staining.

### RNAi and transfection

Freshly disaggregated PDOs were transfected in organoid growing medium with JetPrime Transfection Reagent (Sartorius, Gottingen, Germany) with ctrl- (cat# AM4611) or EpHA2 directed siRNA (cat# AM51331, Thermofisher, Waltham, MA, United States) according to the manufacturer’s instructions, except that the transfection was performed in absence of serum in organoid growing medium as above indicated.

### Aldehyde dehydrogenase activity (ALDH) detection

ALDH activity was assessed by flow cytometry with the ALDEFLUOR kit (Stem Cell Technologies, Vancouver, BC, Canada) following the manufacturer’s instructions, as previously published (Canino et al., 2012; Cioce et al., 2021a). Briefly, the PDO-derived cells, following disaggregation and filtration through a 70 μm mesh, were incubated with BODIPY aminoacetaldehyde, which is converted into a fluorescent molecule (BODIPY-aminoacetate) in the cytoplasm. The specificity of the fluorescence was demonstrated using the specific ALDH inhibitor, diethylaminobenzaldehyde (DEAB). To eliminate dead cells, cells were first stained with the viability dye Sytox-Red (Life Technologies Inc., Grand Island, NY, United States). Cell populations were identified using a Cytoflex flow cytometer (Beckman Colter Life Sciences, IN, United States). The background fluorescence was subtracted from the specific one by using the DEAB-treated samples as a reference.

### Statistics

GraphPad Prism (Version 9.0, https://www.graphpad.com/) was used for data analysis. The data were obtained from at least three independent experiments, unless otherwise indicated, and are presented as the mean ± SEM. Statistical significance between the control and experimental groups was assessed using an unpaired, two-tailed Student’s t-test. A p-value of less than 0.05 was considered statistically significant. The linear regression analysis was performed with https://www.graphpad.com/quickcalcs/linear1/). The Pearson correlation between EphA2 expression and MSI and TMB scores in the Consensus Molecular subtype 1 (CMS1) tumors was performed by employing the K-M plotter (https://kmplot.com/analysis/).

## Data Availability

The original contributions presented in the study are included in the article/Supplementary Material, further inquiries can be directed to the corresponding authors.
