# Supplementary material for: EphA2 sustains the adaptive response of colorectal organoids to chemotherapy
Source: Front Cell Dev Biol. 2026 Jun 12;14:1833389. doi: 10.3389/fcell.2026.1833389 (PMC13303685; doi:10.3389/fcell.2026.1833389)
Supplement: Supplementary file 1 [file Table1.docx]

**Supplementary Table 1.** Histological and clinico-pathological features of the specimens used in this work to generate PDO cultures

| **ID PDO** | | **Histology** | **grade** | **pTNM** |
| --- | --- | --- | --- | --- |
| 1 | adenocarcinoma _right sided | | G2 | pT3, pN1a, Mx |
| 2 | adenocarcinoma _right sided | | G2 | pT3, pN0, Mx |
| 3 | adenocarcinoma _right sided | | G2 | pT3, pN0, Mx |
| 4 | adenocarcinoma _right sided | | G3 | pT4b, pN2b, Mx |
| 5 | adenocarcinoma _right_sided | | G2 | pT3, pN1a, Mx |
| 6 | adenocarcinoma _left_sided | | G3 | pT3, pN0, M1a |
| 7 | adenocarcinoma _left_sided | | G2 | pT3, pN0, Mx |

Legend: T= Tumor; N=nodes: M=metastasis: G=grade
