# Supplementary material for: EphA2 sustains the adaptive response of colorectal organoids to chemotherapy
Source: Front Cell Dev Biol. 2026 Jun 12;14:1833389. doi: 10.3389/fcell.2026.1833389 (PMC13303685; doi:10.3389/fcell.2026.1833389)
Supplement: Supplementary file 2 [file Table2.docx]

**Table S1. List of the main regents and tools employed in this work.**

| REAGENT or RESOURCE | | SOURCE | WORKING DILUTION | IDENTIFIER |
| --- | --- | --- | --- | --- |
|  | Antibodies | | | |
| Human EphA2 Alexa Fluor® 488‑conjugated Antibody Clone # 371805 | | R&D Systems | 3ul/10^5cells | Cat # FAB3035G |
| PE anti-human EphA2 Antibody, Clone # SHM16 | | Biolegend | 1ul/10^5 cells | Cat # 356804 |
| PE Mouse IgG2b, κ Isotype Ctrl Antibody Clone # MPC-11 | | Biolegend | 1ul/10^5 cells | Cat # 400314 |
| Phospho-EphA2 (Ser897) Polyclonal Antibody | | ThermoFisher | 1:1000 in PBS 1%BSA | Cat # PA5-117243 |
| Mouse monoclonal anti-EphA2 Antibody clone # C3 | | Santa Cruz Biotechnology |  | Cat # sc-398832 |
| Mouse monoclonal anti-tubulin antibody clone # C11 | | Santa Cruz Biotechnology | 1:5000 in PBS/1%BSA | Cat # sc-17787 |
| Human TruStain FcX™ | | Biolegend | 1ul/10^5 cells | 422301 |
|  | Chemicals, peptides, and recombinant proteins | | | |
| Oxaliplatin | | Cayman Chemicals |  | Cat # 13106 |
| 5-Fluorouracil | | Cayman Chemicals |  | Cat # 14416 |
| MK2 inhibitor PF-3644022 | | Cayman Chemicals |  | Cat # 19185 |
| Akt inhibitor MK-2206 | | Selleck Chemicals |  | Cat # S1078 |
| IntestiCult™ Plus Organoid Growth Medium | | Stem Cell Technologies |  | Cat # 100-1677 |
| Helix-NP Blue viability dye | | Biolegend |  | Cat # 425305 |
|  | Critical commercial assays | | | |
| RNAeasy minikit | | Qiagen |  | Cat # 74104 |
| Organoid Recovery solution | | The Well Bioscience |  | Cat# MS04-100 |
| High Capacity RNA-to cDNA kit | | ThermoFisher |  | Cat # 4374966 |
| Human EphA2 ELISA Kit | | ThermoFisher |  | Cat # EH173RB |
| JetPrime Transfection Reagent | | Sartorius |  | Cat # 101000015 |
| ALDEFLUOR kit | | Stem Cell Technologies |  | Cat # 01700 |
| SYTOX™ Red Dead Cell Stain | | ThermoFisher |  | Cat # S34859 |
|  | | Oligonucleotides |  |  |
| Cytokeratin 20 (KRT20) Human qPCR Primer Pair (NM_019010) | | OriGene |  | Cat # HP213342 |
| CDX2 Human qPCR Primer Pair (NM_001265) | | OriGene |  | Cat # HP205188 |
| [CD44 Human qPCR Primer Pair (NM_000610)](https://www.origene.com/catalog/gene-expression/qpcr-primer-pairs/hp200577-cd44-human-qpcr-primer-pair-nm-000610) | | OriGene |  | Cat # HP200577 |
| [EPCAM Human qPCR Primer Pair (NM_002354)](https://www.origene.com/catalog/gene-expression/qpcr-primer-pairs/hp206056-epcam-human-qpcr-primer-pair-nm-002354) | | OriGene |  | Cat # HP206056 |
| Silencer™ Negative Control No. 1 siRNA | | ThermoFisher |  | Cat # AM4611 |
| Silencer™ EphA2 siRNA | | ThermoFisher |  | Cat # AM51331 |
|  | Software | | | |
| GraphPad Prism Version 9.0 | | https://www.graphpad.com/updates/prism-900-release-notes | | |
| KM plotter | | https://kmplot.com/analysis/ | | |
| NIH Image J | | https://imagej.net/ij/index.html | | |
