# Supplementary figures and images for: EphA2 sustains the adaptive response of colorectal organoids to chemotherapy
Source: Front Cell Dev Biol. 2026 Jun 12;14:1833389. doi: 10.3389/fcell.2026.1833389 (PMC13303685; doi:10.3389/fcell.2026.1833389)

# Figure S1

a

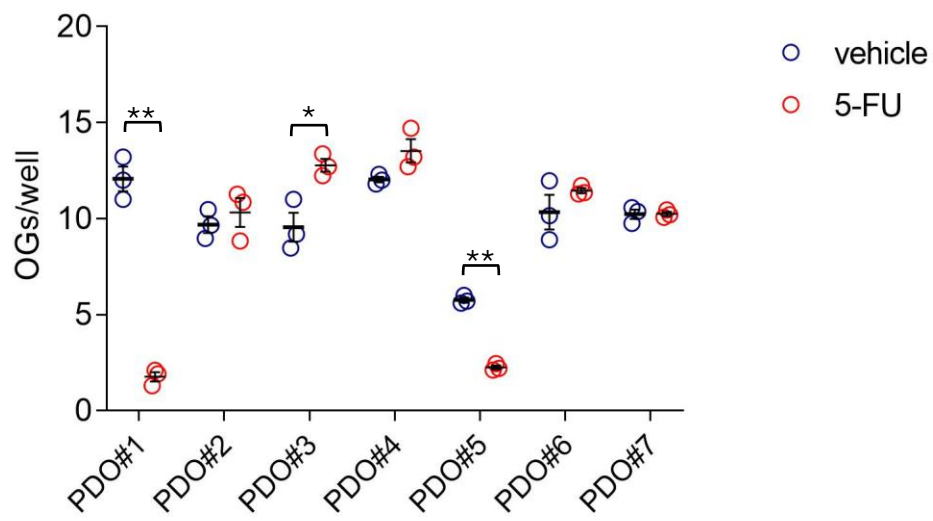

b

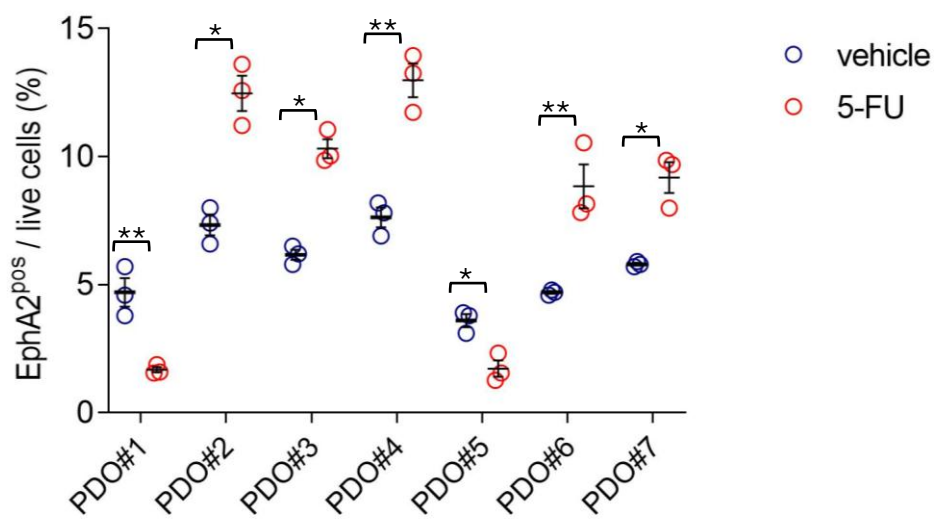

c

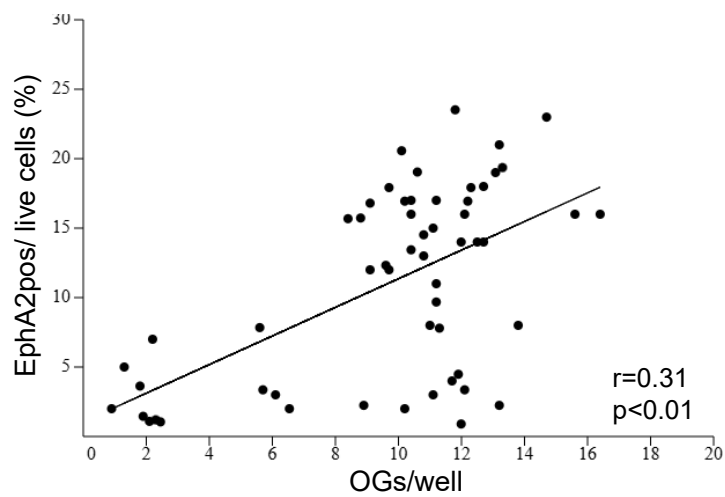

Figure S2

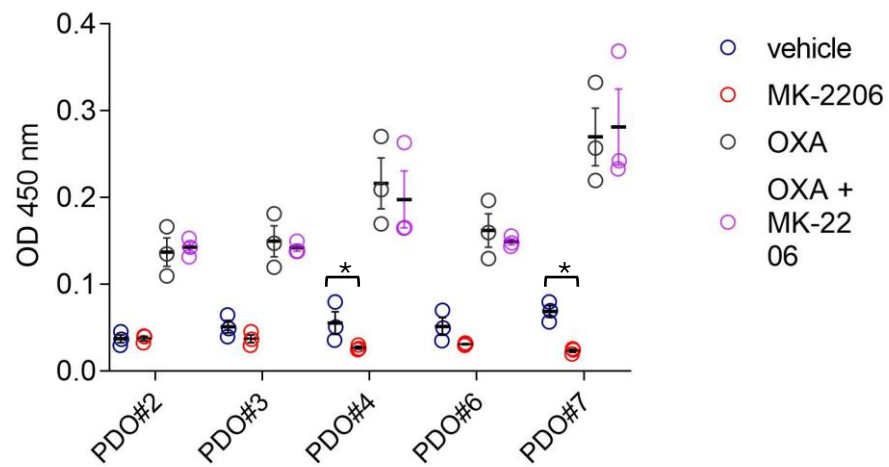

Figure S3

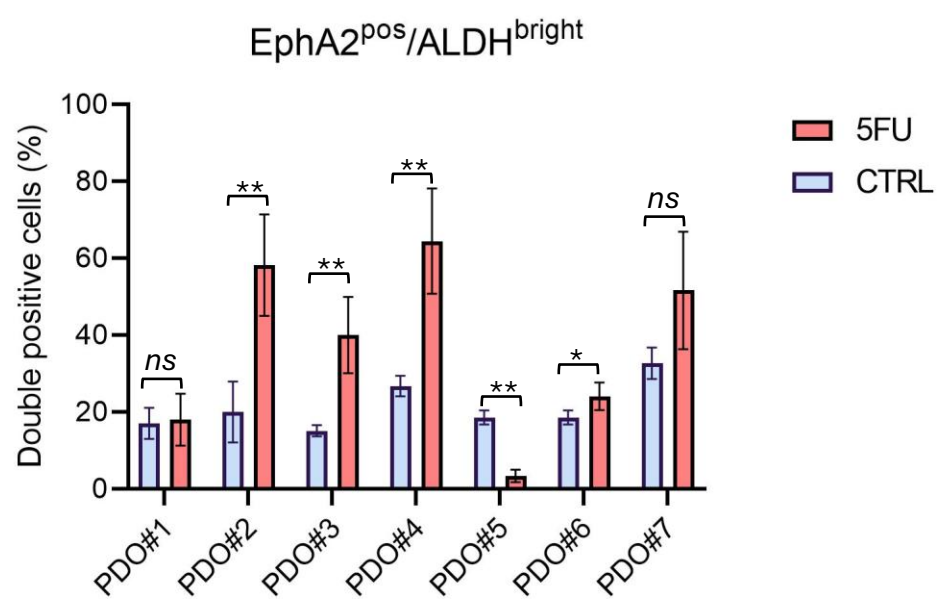

Supplement: Supplementary file 3 [file DataSheet1.pdf]
